# High Order Profile Expansion to tackle the new user problem on recommender systems

**DOI:** 10.1371/journal.pone.0224555

**Published:** 2019-11-07

**Authors:** Diego Fernández, Vreixo Formoso, Fidel Cacheda, Victor Carneiro

**Affiliations:** Center for Information and Communications Technology Research (CITIC), Department of Computer Science and Information Technologies, University of A Coruña, A Coruña, Spain; Vietnam National University, VIET NAM

## Abstract

Collaborative Filtering algorithms provide users with recommendations based on their opinions, that is, on the ratings given by the user for some items. They are the most popular and widely implemented algorithms in Recommender Systems, especially in e-commerce, considering their good results. However, when the information is extremely sparse, independently of the domain nature, they do not present such good results. In particular, it is difficult to offer recommendations which are accurate enough to a user who has just arrived to a system or who has rated few items. This is the well-known new user problem, a type of cold-start. Profile Expansion techniques had been already presented as a method to alleviate this situation. These techniques increase the size of the user profile, by obtaining information about user tastes in distinct ways. Therefore, recommender algorithms have more information at their disposal, and results improve. In this paper, we present the High Order Profile Expansion techniques, which combine in different ways the Profile Expansion methods. The results show 110% improvement in precision over the algorithm without Profile Expansion, and 10% improvement over Profile Expansion techniques.

## Introduction

Available information is growing every day. Many e-commerce sites have an overwhelming number of items to offer. Due to this, they use Recommender Systems to present suitable information according to user tastes, simplifying the decision making process. Consequently, users are more likely to find what they are looking for and satisfy their needs, which, at the same time, leads to a potential increase in the sites profits.

Among the recommendation techniques, Collaborative Filtering [1] has shown very good results in many domains. They build profiles based on user preferences. Using these profiles they search for similarities between users or items, in order to accomplish different recommendation tasks [2]. However, when a particular user has very few ratings, the system is not capable of computing a recommendation good enough for them, since it does not know enough about their tastes [3]. This is the well-known cold start problem [4] and, more concretely, the so-called new user problem [5].

In this work we explore the Profile Expansion (PE) techniques [6], which were proposed to deal with the new user problem without involving the user. They automatically provide the recommender algorithm with more information about user tastes. In fact, the nature of this information highly depends on the technique chosen. We analyze their behavior in several situations and we expose their benefits and limitations. Considering this analysis, we propose how to combine these techniques to take advantage of their benefits, while minimizing the impact of their limitations. This way the user profile is expanded with rating information obtained from diverse sources. The new methods defined are called High Order Profile Expansion (HOPE) techniques, since instead of just using one PE technique, two or more techniques can be combined so that new and diverse ratings are added to user profiles with few ratings.

In this paper we aim at demonstrating how the results obtained by PE techniques are improved by combining different PE techniques. To do so, we will compare the accuracy of the recommendations obtained by a user-based k-Nearest Neighbors (kNN) algorithm [7] in a new user cold-start situation without PE, computing simple PE and applying HOPE.

In the next section we discuss the related work that tackles the new user problem. We especially focus on PE techniques. Following, we explain our proposed method and its variations. Next, we lay out the experiments we have performed and the results are discussed. Finally, we present some conclusions and the future work.

## State of the art

Collaborative Filtering techniques are only based on user preferences in the form of item ratings in order to make a recommendation. Among them, the kNN algorithms are very popular [7] because they are simple, stable, easy to explain [8] and they can make the recommendation in real-time. This is desirable for online commercial sites and social networks [9]. They work by finding a set of the k users most similar to the user they are computing a recommendation for (the active user). These k users are known as neighbors. The similarity is computed according to a similarity function. Pearson correlation [10] and cosine vector similarity [11] are commonly used.

The kNN algorithms can be classified into user-based [10] and item-based [12]. When a user-based kNN algorithm is used (similarly with item-based) users rely on the opinion of their k most like-minded people (their neighbors) in order to obtain an accurate recommendation. Either a rating prediction for a particular item (annotation in context task), or a recommendation list (find good items task) [2], is obtained from the information provided by these neighbors.

Collaborative Filtering algorithms work well when many ratings are available. However, sometimes the information about a particular user (new user problem), item (new item problem) or, even, the information in the whole system (new system problem) is really scarce. These are the three cold-start situations [4].

In this work we will focus on the new user problem. In the literature different approaches have been used to address this problem. Basically, they can be divided into those where the user is involved to provide the system with additional information and those without user intervention.

The easiest way to obtain additional information about a user is to ask them directly for it. The process of gathering these initial ratings, known as the elicitation process [13], can mean the success or the failure in keeping a user in the system [14]. The initial items should be carefully chosen and the number of selected items should not be excessive to avoid bothering the users [15]. In exchange, users expect to receive good recommendations. This way, every user will have at least the number of ratings that the system requests while signing up. Different solutions have been proposed to deal with this elicitation process. Rashid *et al*. study how to learn new preferences applying decision theory and entropy [13]. Other authors use a tree-based bootstrapping process to elicit users to provide their opinions [16], a lazy decision tree, with pair-wise comparisons at the decision nodes [17] or even gamification [18]. As it was previously mentioned, the number of ratings that should be collected from a new user before providing recommendations becomes a key aspect to study [15].

However, sometimes it is not adequate to ask the user directly. For example, these requests may annoy some users who consider it is not necessary to rate items when they have just arrived to the system. When a user has few ratings the recommendation is usually inaccurate or the system can be incapable of computing the recommendation. As such, and depending on the context, it may be even better not to show recommendations than to bother the users. In any case, if the information is scarce, but the system can provide a user with recommendations accurate enough to keep them in the system, the elicitation process may be unnecessary.

Other alternatives without user intervention are preferable. For example, in [19] the authors show how recommendations biased by popularity, recency and positive ratings do not suit all new users and therefore explore user coverage to diversify recommendations. Some authors studied how to modify kNN algorithms to improve the recommendations. Traditional similarity measures present several problems when the information is scarce because they are too strict [20]. Several similarity measures have been presented as an alternative to the traditional ones in these situations: a new similarity measure for kNN algorithms, based on proximity, impact and popularity of user ratings [21]; another one based on the combination of six individual similarity measures [22]; or a probability-based similarity measure [23].

In addition to this, PE techniques have been presented as a method to deal with the new user problem without user intervention [6]. They are based on Query Expansion techniques used in Information Retrieval [24]. In particular, they increase the information available for a user-based kNN algorithm, which accomplishes the find good items task, that is, it tries to find a list of items that can be to the user taste. Following, we explain these techniques and the research results more in detail.

### Profile Expansion techniques

In Collaborative Filtering a user profile is comprised of the items that the user has rated, together with the ratings they gave to these items. When a user profile is very small, that is, when there is very scarce information about the user, the similarity measures do not usually work well, so the neighborhood is not computed correctly. Since the information for computing the recommendation list is obtained from the neighborhood, the recommendation accuracy will be low. Therefore, user-based kNN algorithms do not work properly in a new user situation [25].

Profile Expansion techniques provide the recommender algorithm with more information about the user. So as to do this, they increase the user profile with additional items, being *l* the number of items added. Moreover, they do it automatically, without involving the user.

**Fig 1:**
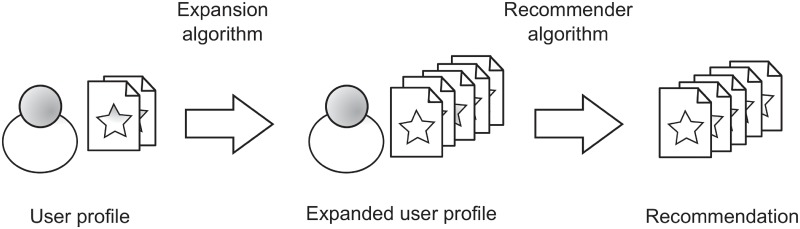
Recommendation process using Profile Expansion.

Fig 1 shows the recommendation process when PE is used. First, the initial user profile is expanded by means of an expansion algorithm. The resulting profile is the input for the recommender algorithm, which is in charge of computing the recommendation list. Particularly, as it was already mentioned, a user-based kNN has been used as the main recommender algorithm, although other algorithms could have been used, too.

Different approaches have been proposed for expanding the user profile. We focus on collaborative techniques, since they take advantage of the existing user profile and they do not require the user to provide extra information. Among them, item-global (IG) and user-local (UL) techniques showed the best results [6].

IG techniques expand the user profile by searching in the system for the most similar items to those rated by the user. They are applied before computing the neighborhood for the main algorithm. An item-based kNN algorithm has been used for that. The recommendation list obtained by means of these techniques has low specificity because global information is used; so, the items added to the expanded user profile will have low specificity. However, since most items that are similar to those in the user profile are likely to be chosen by the algorithm (by taking into account all the items in the system), these techniques do not usually make many mistakes. That is, most items in the recommendation list (not only those that are on top) are frequently related to the user profile items.

On the other hand, UL techniques only use the neighborhood computed for the main algorithm as a source of information to find new items. Once these new items are obtained, and, therefore, the expanded profile, the main algorithm computes a new neighborhood in order to present the recommendation list. For the item selection, several techniques were proposed. Most rated (MR) approach selects the most rated items in the neighborhood. Local item neighbors (LN) and user-local clustering (LC) attempt to find similarities between items. Whereas the former only takes into account the items in the neighborhood, LN and LC compute similarities between the neighborhood ratings to the items in the active user profile and also to the remaining items in the system. With UL algorithms, the most similar items will probably be found, because neighbors will tend to rate them. However, all the items in the neighborhood are not usually related to those in the active user profile. So, the most similar items to those in the user profile will be on the recommendation list, but items unrelated to user tastes can also appear on this list.

In summary, PE techniques deal with the new user problem. As said, collaborative PE techniques have shown promising results [6]. They provide user profiles with more information in order to mitigate the flaws that traditional similarity measures for kNN algorithms present when few ratings are available. Moreover, user profiles have been expanded with collaborative filtering techniques, so users do not have to supply additional information to the system. In any case, this advantage could be suppressed and algorithms using any other source of information could be used to expand the user profiles, such as content-based or demographic techniques, for instance.

However, it is important to note that when items unrelated to those in the user profile are chosen, PE techniques may induce errors. In this regard, predicted ratings for those items unconnected with user profile items will not usually be accurate enough, since the expansion algorithm lacks information to predict their ratings properly. Furthermore, the recommender algorithm will also have more difficulties when finding suitable neighbors who share a minimum number of items in common with the active user, because unrelated items tend to appear less frequently than related items in a same user profile. In the next section we present the HOPE techniques, which try to minimize these errors.

## High Order Profile Expansion

Every PE technique presented in the previous section has its own pros and cons. The idea of HOPE is to combine these techniques to take advantage of their benefits and minimize their errors. Using more than one algorithm to expand the user profile, the expansion will be more heterogeneous and, at the same time, still related to the initial user profile. The aim is still to expand the user profile in order to improve recommendations when facing the new user problem. For the sake of clarity, we will focus on combinations of two algorithms (order two combinations), although it can be extrapolated to any order combinations. Also note that the proposed models could be applied to any user-based kNN algorithm.

The aim of HOPE alternatives is to minimize the errors committed when choosing the items to expand and inferring their corresponding ratings. As the number of items in the expansion increases, the probability of error grows, too. Unlike PE techniques alone, HOPE involves that every algorithm selects few items, which are likely to be related to the user taste.

In our experiments, we will focus on IG and UL PE alternatives because they represent the best PE techniques [6]. These techniques will be combined according to two different proposals: serial and parallel. These proposals determines how the information flows between the algorithms.

### Serial

**Fig 2:**
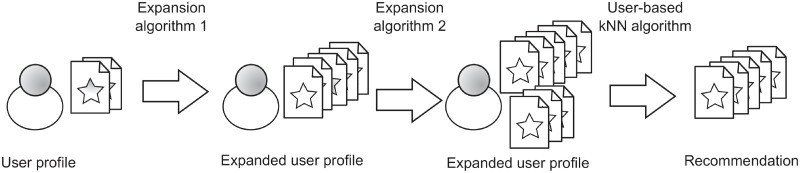
Recommendation process using High Order Profile Expansion with the serial alternative.

In a serial process PE techniques are used one after the other. That is, they are executed as a pipeline, where the output of the first expansion algorithm is the input of the second one (as shown in Fig 2). The kNN algorithm will use the doubly expanded profile to compute the recommendation list. So, serial combination can be considered as the closest way to the PE original concept, where one algorithm is used for increasing the information available for another algorithm. The first algorithm should work properly and minimize errors when the information available is very scarce, because an error in this first step is carried along the remaining phases.

Given that we are dealing with an expansion in two steps, there is an alternative where the same algorithm is applied twice. The difference between having a baseline algorithm with a profile expansion size and having the same algorithm executed twice in a serial expansion process with half the size is that items in last positions of the expansion list computed with the baseline algorithm are more likely to be non-relevant and, then, lead to worse recommendations. As profile expansion size increases, the probability of finding non-relevant items at last positions is higher. Therefore, a priori, a serial process will be preferable with the same algorithm executed twice than the baseline algorithm alone with a double PE size.

### Parallel

**Fig 3:**
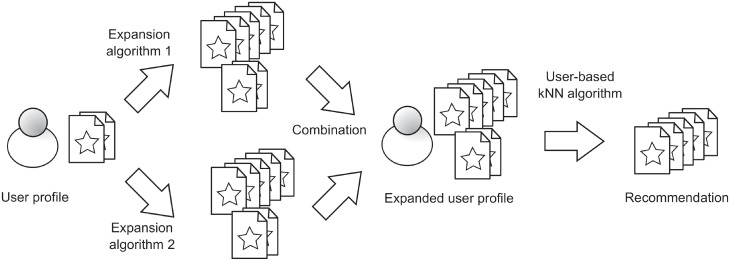
Recommendation process using High Order Profile Expansion with the parallel alternative.

As said, PE techniques may induce errors in item selection and rating prediction, which can affect the final recommendation. This approach attempts to minimize these errors, being the initial user profile the input for all the expansion algorithms used, as it is shown in Fig 3.

Since every expansion algorithm will obtain a new profile and the kNN technique needs a unique profile as input, it is necessary to combine the different profiles. In this combination, we must consider two aspects: rating and item selections. The expanded profiles are obtained independently, so they may or may not have items in common. Regarding the item selection, two alternatives have been considered:

- Union. The set of items in the final user profile is comprised of the union of the items in every expansion. Therefore, items that appear in only one expanded profile will also be selected. Logically, when combining the same algorithm more than once, the result is the expansion with the biggest PE size, *l*.
- Intersection. The set of items in the final user profile is comprised of the intersection of the items in every expansion. That is, only items appearing in all the expanded profiles will be part of the resulting user profile. In the case of combining the same algorithm more than once, this time the result is the expansion with the smallest *l*.

As far as rating selection is concerned, when an item appears in more than one expanded profile, the final rating will be the mean of the ratings.

In parallel combination, unlike serial approach, when an error is committed by an expansion algorithm, this error does not affect the expansion computed by the other algorithm. In exchange, the second algorithm has at its disposal less information, since it only has the initial user profile as input.

## Experiments

### Experiment design

The experiments have been performed in a movie recommendation domain. Particularly, we have used a reduced version of the Netflix dataset [26], where each item matches a movie from the Internet Movie Database (IMDb). This way, we can also do a human evaluation of the results and, in the future, content-based algorithms can be compared, too. The final dataset consists of 8,362 items and 478,458 users, who have done 48,715,350 ratings between 1 and 5 both inclusive.

Regarding the methodology, we have randomly selected 1000 users for evaluation purposes. The new user problem has been simulated using a Given-*N* strategy [11]. That is, *N* ratings have been randomly selected as part of the training subset. The remaining ratings take part in the evaluation subset. We focus on *N* = 2, since it is when the information is more scarce and HOPE techniques can be more useful. Moreover, we study the evolution of the algorithms according to *N*. From the remaining users, we have randomly chosen 90% of ratings for the training subset.

In relation to the algorithms, we first study how item-global and user-local techniques behave alone, since they are the PE algorithms that obtained the best results in [6]. Then, we tackle the different ways to combine them. The main algorithm is a user-based kNN algorithm, as was previously mentioned. The number of neighbors, *k*, is 25, selected after a cross-validation process.

Since we focus on the find good items task, a list of recommended items is returned by the algorithm. For evaluating the results, we consider 4 as the relevance threshold. We have used traditional metrics such as precision and MAP. In particular, Precision-at-5 (P@5), precision at the 5 first positions of the recommendation list, is especially interesting, because it considers those items the user is likely to pay attention to, and not all the list, as MAP does.

Throughout this section only the most significant results will be shown. In fact, we also report on statistical significance using a standard one-sided t test with significance level alpha = 0.05 when improving performance of the different baseline models considered.

### Basic Profile Expansion techniques

Table 1 shows the P@5 results with different *l* (profile expansion sizes). As discussed in the Profile Expansion section, an item-based kNN algorithm is employed as IG technique. We have used cosine vector similarity for computing the neighborhood. For the IG method, P@5 results improve as *l* is smaller. However, between *l* = 2 and *l* = 5 there is no significant difference. In fact, MAP results for *l* = 5 are better.

**Table 1:** P@5 and MAP for Profile Expansion baseline algorithms. Significant improvements over the algorithm without PE are highlighted.

Algorithms	*l*	*N* = 2		*N* = 10	
		P@5	MAP	P@5	MAP
IG	2	**0.125**	0.029	0.123	**0.040**
	5	**0.122**	**0.035**	0.118	**0.045**
	10	**0.100**	**0.038**	0.097	**0.046**
	15	0.086	**0.039**	0.076	**0.044**
	20	0.078	**0.038**	0.071	**0.043**
	50	0.044	0.034	0.048	0.037
LC	2	**0.136**	0.025	0.148	0.035
	5	**0.140**	0.030	0.140	0.036
	10	**0.123**	**0.034**	0.128	**0.040**
	15	**0.112**	0.**036**	0.118	**0.041**
	20	**0.102**	**0.038**	0.110	**0.042**
	50	0.077	**0.040**	0.085	0.039
LN	2	**0.134**	0.023	0.140	0.037
	5	**0.139**	0.029	0.141	0.039
	10	**0.128**	0.032	0.140	0.038
	15	**0.121**	**0.035**	0.141	0.037
	20	**0.111**	**0.037**	0.138	0.037
	50	0.083	**0.041**	0.137	0.037
MR	2	**0.138**	0.024	0.146	0.031
	5	**0.147**	0.029	0.139	0.035
	10	**0.134**	**0.032**	0.127	0.038
	15	**0.117**	**0.033**	0.123	0.037
	20	**0.109**	**0.036**	0.115	0.038
	50	0.076	**0.040**	0.087	**0.042**
No PE	-	0.078	0.029	**0.140**	0.036

On the other hand, Table 1 also shows the P@5 results for the best UL algorithms presented in [6]: most rated (MR), local item neighbors (LN) and user-local clustering (LC). Despite the fact that the MR technique only considers the most rated items in the neighborhood and LN and LC techniques try to capture similarities between local items and other items in the system not being in the neighborhood, all these techniques present similar results in P@5. Although it does not present such good results with the MAP metric, the MR technique is preferable because of its simplicity. For sake of completeness, Table 1 shows the performance without using Profile Expansion techniques (row tagged as No PE).

Considering these results, PE techniques with *l* = 5 will be used as the basic algorithms for analyzing the HOPE techniques. In addition, we will consider the MR algorithm and the algorithm without PE as the baselines when using *N* = 2 and *N* = 10, respectively. In particular, the p-value obtained for the MR algorithm (*l* = 5) when analyzing the statistical significance of the results has been 1.44e-18.

### High Order Profile Expansion alternatives

Among the different HOPE alternatives, we are interested in evaluating which one behaves best and in knowing their pros and cons. In order to achieve that, different experiments have been evaluated. Regarding profile expansion size variations, we have used different *l* values (2, 5, 10 and 15) to check how the *l* combinations affect the results. Moreover, it is also interesting to know how the distinct algorithms evolve according to *N*, that is, according to the amount of information available.

#### Serial

In serial combinations, two Profile Expansion techniques are used, one after the other. Although algorithms in the previous section obtain their best results with *l* = 5, the best results combining these algorithms in a serial process are obtained with *l* = 2, reinforcing the discussion presented in the Serial HOPE section. In fact, the PE algorithms also present very good results with this profile expansion size.

Table 2 shows P@5 and MAP results for *l* = 2 combinations. Results for *l* values of 5, 10 and 15 are omitted due to their lower performance.

**Table 2:** P@5 and MAP for High Order Profile Expansion serial combinations, with *l* = 2. Results for simple Profile Expansion techniques are also shown, with *l* = 5. Significant improvements over the MR algorithm (when *N* = 2) and the algorithm without PE (when *N* = 10) are highlighted.

Algorithms		*N* = 2		*N* = 10	
1	2	P@5	MAP	P@5	MAP
LC	LC	0.153	0.026	0.146	0.034
	LN	0.147	0.026	0.148	0.036
	MR	0.156	0.025	0.153	0.036
	IG	0.149	**0.032**	0.147	**0.040**
	-	0.140	0.030	0.140	0.036
LN	LC	0.154	0.027	0.151	0.035
	LN	0.142	0.024	0.140	0.037
	MR	0.158	0.025	0.151	0.033
	IG	0.156	**0.032**	0.127	**0.041**
	-	0.139	0.029	0.141	0.039
MR	LC	**0.163**	0.026	0.153	0.034
	LN	0.152	0.025	0.146	0.032
	MR	0.155	0.024	0.151	0.034
	IG	0.160	0.030	0.137	0.039
	-	0.147	0.029	0.139	0.035
IG	LC	0.139	0.030	0.144	**0.040**
	LN	0.137	0.031	0.128	**0.041**
	MR	0.145	0.029	0.143	**0.041**
	IG	0.133	**0.035**	0.129	**0.045**
	-	0.122	0.035	0.118	**0.045**
No PE		0.078	0.029	0.140	0.036

Unlike parallel alternatives, in this case the execution order may be important. So, first, we will focus on algorithm order. Regarding the results, the LN algorithm works better when it is used as the first algorithm. Moreover, the IG algorithm performs better when it is the second algorithm, significantly improving the MAP values with respect to the baselines. The MR and LC algorithms depend on which algorithm is combined with them. These observations are predictable and logical, because when the information about a user is scarce, global information is more prone to errors, since it is more difficult to select which items are really similar to those in the user profile. However, when working with local information, although there is less information available, it tends to be related to the user profile. From now on, when two algorithms can be combined using two different orders, we will only present the order that performs better.

In order to deal with the different combinations, we will divide them into three groups: first, those that combine IG with any other algorithm; second, those that combine two different UL algorithms; and, third, those that combine an algorithm with itself.

**Fig 4:**
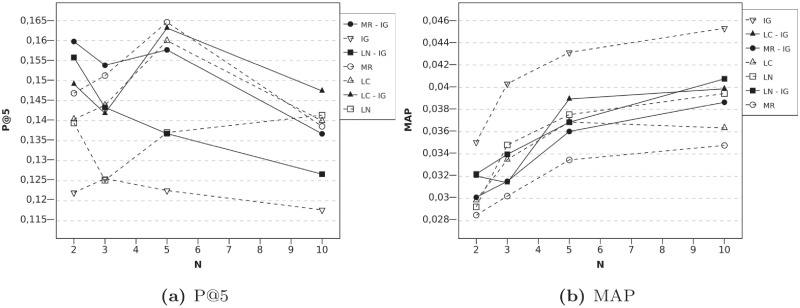
Evolution according to N, High Order Profile Expansion using serial combinations with IG technique and *l* = 2. Fig (a) reports P@5 results and (b) shows MAP.

Fig 4 shows P@5 and MAP results when user-local techniques are combined with the IG technique. When *N* = 2, that is, when the available information about the active user is extremely scarce, this kind of combinations improves the precision baselines. In particular, combining the MR algorithm with the IG algorithm improves the results almost 10%. Moreover, MAP results regarding MR improve, too. Since they use different types of information, when using both algorithms, the recommendation list can be more accurate, because more information about the user is obtained. In addition to this, and for the same reason, if LN is the algorithm that is combined, then the improvement in P@5 is greater than using LN and IG algorithms, when *N* is low.

The behavior of the MR algorithm and LN algorithm with IG approach is very similar. The precision tends to diminish according to the increase of *N*. This is because the scarcity of information is not as critical when *N* = 10. In fact, whereas the algorithm without PE can obtain a precision of about 0.14, High Order alternatives can induce several errors in rating predictions, which can lead to a loss of accuracy in the recommendation.

**Fig 5:**
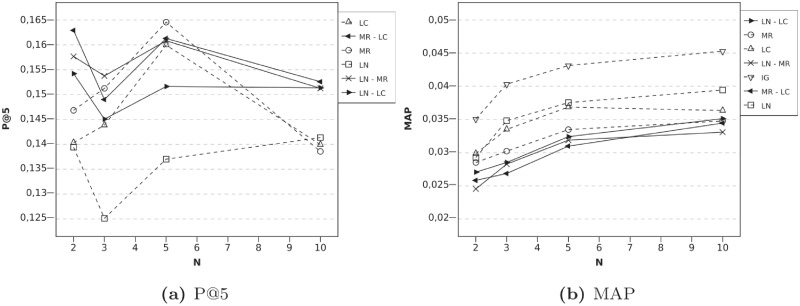
Evolution according to N, High Order Profile Expansion using serial combinations with UL techniques and *l* = 2. Fig (a) reports P@5 results and (b) shows MAP.

Regarding the serial combination of UL algorithms, they also present good results, as can be seen in Fig 5. Again, it is with *N* = 2 when we obtain the greatest improvement, since the usefulness of PE techniques increases when very little information about the user is available. Given that the MR algorithm originally had high precision, combinations with this algorithm reach high precision, too. The combination with LC obtains the best P@5 result and significant improvements over the MR algorithm performed alone (the p-value was 0.04). In this case, good results are also achieved when *N* = 10. However, MAP values obtained are worse than those reached when IG techniques participate in the combinations. IG techniques employ global information, so most items used for expansion will be close to user tastes, whereas using UL techniques can imply that some items not so similar to user tastes are incorporated to the user profile. Nevertheless, as was mentioned previously, in general, we consider P@5 more valuable than MAP for the find good items task, although it depends on the particular context of each system.

**Fig 6:**
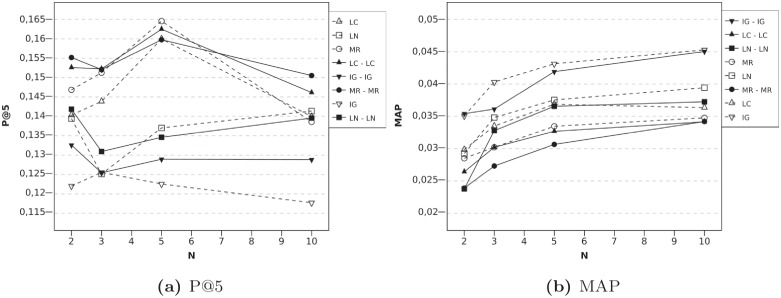
Evolution according to N, High Order Profile Expansion using the same algorithm twice and *l* = 2. Fig (a) reports P@5 results and (b) shows MAP.

Finally, an algorithm can also be combined with itself. The results are shown in Fig 6. All the algorithms, but LN algorithm, improve the precision results when they are executed in a serial process. These results confirm that a serial process is preferable to the baseline algorithm with double the profile expansion size, as was mentioned in the Serial HOPE section. MAP results are only slightly lower than those obtained with the baseline algorithms.

As a general result, the serial combinations of UL algorithms show very good results in P@5, whereas the combinations with IG techniques present better results in MAP. Using the MR algorithm with either LC or IG techniques is particularly interesting, since these combinations have a 10% improvement in P@5 over UL algorithm results and around 110% improvement over the algorithm without PE. In fact, the combination of MR approach with any other alternative always improves the baselines results. The reason is that they complement each other, since they use different sources of information.

#### Parallel

The parallel variants presented in this paper are union and intersection. Table 3 shows P@5 and MAP results for both alternatives. Note that as union and intersection operations are commutative, only one combination is presented for each pair of algorithms.

**Table 3:** P@5 and MAP for High Order Profile Expansion parallel alternatives. Intersection alternative with *l* = 5 for all the algorithms but IG, with *l* = 100. Union alternative with *l* = 2 for all the algorithms. Results for simple Profile Expansion techniques are also shown, with *l* = 5. Significant improvements over the MR algorithm (when *N* = 2) and the algorithm without PE (when *N* = 10) are highlighted.

Combinations	Algorithms		*N* = 2		*N* = 10	
	1	2	P@5	MAP	P@5	MAP
Intersection	IG	LC	0.128	0.029	0.148	0.038
		LN	0.139	0.028	0.141	0.039
		MR	0.133	0.027	0.143	0.038
	LC	LN	0.146	0.029	0.141	0.038
		MR	0.143	0.029	0.152	0.036
	LN	MR	0.147	0.028	0.141	0.038
Union	IG	LC	0.142	0.031	0.140	**0.041**
		LN	0.134	0.030	0.125	**0.040**
		MR	0.144	0.030	0.148	**0.040**
	LC	LN	0.137	0.025	0.147	0.034
		MR	0.144	0.027	0.152	0.034
	LN	MR	0.138	0.024	0.148	0.032
Simple PE	LC	-	0.140	0.030	0.140	0.036
	LN	-	0.139	0.029	0.141	0.039
	MR	-	0.147	0.029	0.139	0.035
	IG	-	0.122	0.035	0.118	0.045
No PE	-	-	0.078	0.029	0.140	0.036

Intersection alternative consists in intersecting expanded profiles obtained with different techniques in order to create a unique expanded profile. These techniques can be of different nature. Because of that, their expanded profiles may have no items in common if we take into account very few items as expansion. This could cause the Profile Expansion step to become useless. Therefore, for this variant profile expansion sizes have been increased with respect to the serial alternative, so that it is more probable that two different techniques have items in common.

While *l* = 5 is enough for obtaining good results when only UL algorithms are considered, the same does not occur when IG techniques are used. In fact, in the whole dataset there can be many items similar to those in the user profile and only a few of these are likely to appear as items rated by user neighbors. This is the reason why we decided to use a bigger profile expansion size for IG algorithm (*l* = 100). Indeed, our results confirmed this assumption.

From Table 3, we can observe that MAP values are quite good, but P@5 results for the parallel variation do not improve those obtained with the serial alternative.

On the other hand, union variation puts together the expansions obtained with the different algorithms. We have used *l* = 2 as profile expansion size. As shown in Table 3 the results do not improve those obtained with the serial alternative either. In fact, no combination gets a P@5 value better than MR baseline algorithm.

So, despite the fact that with the parallel alternatives the second expansion algorithm is not affected by the errors committed by the first one, the serial results are better because the second algorithm is provided with more information.

## Conclusions and future work

In this work, we study HOPE techniques in depth, which deal with the new user problem in Collaborative Filtering. In particular, we propose two methods that combine basic PE techniques to take advantage of their benefits and mitigate their errors. These methods are called serial and parallel.

The experiments have shown how the serial alternative performs better than the parallel one. The reason is that in the serial alternative an algorithm is supplied with the information obtained by another algorithm. Without it, the second algorithm may not obtain this information. That does not happen with the parallel alternative, where the algorithms use the same information.

Regarding the different serial combinations, those where the MR algorithm takes part obtain the best P@5 results. Moreover, the IG algorithm increases MAP obtained with UL algorithms when they are used together. This way, the combination of IG and MR methods performs very well, mainly when *N* is low.

Furthermore, when two different UL methods are combined, the results in P@5 reach 0.145 with all the values studied for *N*. However, MAP values do not surpass those obtained by the MR method, the lowest among all the UL alternatives.

Another way to combine PE techniques is to execute the same algorithm two consecutive times. In this case, all the algorithms, but LN method, improve the results obtained when they are executed only once.

In the future, we plan to evaluate how a content-based algorithm behaves in combination with other PE algorithms, since, throughout this paper, only item-based kNN algorithm has been used as IG method. Other Collaborative Filtering algorithms apart from neighbor-based ones could also be used and tested. In fact, we plan to combine more than two different algorithms, since in this work we have analyzed the two by two combinations. Finally, we are also interested in analyzing how these techniques behave in a new system situation.

## Supporting information

S1 Dataset Dataset for the High Order Profile Expansion. The complete dataset for the High Order Profile Expansion experiments can be accessed via: https://doi.org/10.6084/m9.figshare.9798155.
(PDF)
