# Supplementary material for: High Order Profile Expansion to tackle the new user problem on recommender systems
Source: PLoS One. 2019 Nov 7;14(11):e0224555. doi: 10.1371/journal.pone.0224555 (PMC6837286; doi:10.1371/journal.pone.0224555)
Supplement: S1 Dataset — The complete dataset for the High Order Profile Expansion experiments can be accessed via: https://doi.org/10.6084/m9.figshare.9798155. (PDF) [file pone.0224555.s001.pdf]

Supporting information

S1 Dataset.

Dataset for the High Order Profile Expansion.
The complete dataset for the High Order Profile Expansion experiments has been published in the public repository: https://doi.org/10.6084/m9.figshare.9798155
